# Spin-based Optomechanics with Carbon Nanotubes

**DOI:** 10.1038/srep00903

**Published:** 2012-11-29

**Authors:** Jin-Jin Li, Ka-Di Zhu

**Affiliations:** 1 Key Laboratory of Artificial Structures and Quantum Control (MOE), Department of Physics, Shanghai Jiao Tong University, 800 Dong Chuan Road, Shanghai 200240, China

## Abstract

A simple scheme for determination of spin-orbit coupling strength in spinbased optomechanics with carbon nanotubes is introduced, under the control of a strong pump field and a weak signal field. The physical mechanism comes from the phonon induced transparency (PIT), by relying on the coherent coupling of electron spin to vibrational motion of the nanotube, which is analogous to electromagnetically induced transparency (EIT) effect in atom systems. Based on this spin-nanotube optomechanical system, we also conceptually design a single photon router and a quantum microwave transistor, with ultralow pump power (~ pW) and tunable switching time, which should provide a unique platform for the study of spin-based microwave quantum optics and quantum information processing.

Carbon nanotubes (CNTs) provide a number of attractive features, including low masses, high-Q factors, large confinement energies and nearly nuclear-spin-free environment, which made them interesting candidates for the development of ultrasensitive detection and quantum information science^1^,^2^,^3^. Remarkably, the investigation of spin-orbit interaction (SOI) in CNTs has recently become an active research field for the special case of one and two carriers in ultraclean CNT, which provide a route for manipulating the spin degree of freedom^1^,^4^,^5^. Understanding the coupling strength of SOI in different situations is central for applications in spin-based nanodevices and quantum computations. Recently, Kuemmeth *et al.*^6^ and Burkard *et al.*^7^ have studied the coupling of spin and resonator (phonon “cavity”) modes in suspended CNT in laboratory and in theory, respectively, for the cases where the spin-resonator coupling arises from the inherent spin-orbit interaction. The former reported the measurements that in clean nanotubes the spin and orbital motion of electrons are coupled, while the later predicted that strong spin-orbit coupling can be realized with current state-of-art devices. Here we shall theoretically propose a simple scheme to realize the measurement of spin-orbit coupling strength in spin-based optomechanics with carbon nanotubes, where the electron spin is interacted with the vibrational motion of CNT due to curvature-induced spin-orbit coupling.

Besides, the conventional optomechanical system has been appointed as the boundary between classical and quantum mechanical system, which consists of an optical cavity coupled to a mechanical oscillator via radiation pressure^8^,^9^,^10^,^11^. Substituting of mechanical element or optical cavity to other nanometer or micrometer scale system has the possibility to inherit the properties of cavity optomechanics and develop advantages of new materials^12^. In the presence of a strong pump field and a weak signal field, the coupling of electron spin to vibrational motion in suspended carbon nanotube is analogous with the coupling of cavity to mechanical resonator in optomechanical system, where the role of the optical cavity is played by an electron in the presence of external magnetic field, while the role of the mechanical element is replaced by the CNT's vibration.

We further conceptually design a single photon router and a quantum microwave transistor based on the spin-CNT optomechanical system due to the phonon induced transparency. Both of them are operated at ultralow pump power and tunable switching time, which will make a contribution for spin-based quantum information processing. For a single photon router, the signal photons would be reflected by the spin-CNT system in the absence of the pump field. Otherwise, if turning on the pump field and fixing the pump frequency on resonance with the frequency of the CNT vibration mode and the qubit simultaneously, the signal photons are transmitted due to the phonon induced transparency, which is analogous to electromagnetically induced transparency effect in atom systems^13^,^14^. In this case, which channel of the router is selected depends on a tunable pump field. Furthermore, as a quantum microwave transistor, the pump field not only has a switch behavior to choose the status of output signal (reflected or transmitted), but also play a role to amplify the output signal field, which is essential to the quantum information processing with photons as signal carrier other than the electrons.

## Results

**Figure 1:**
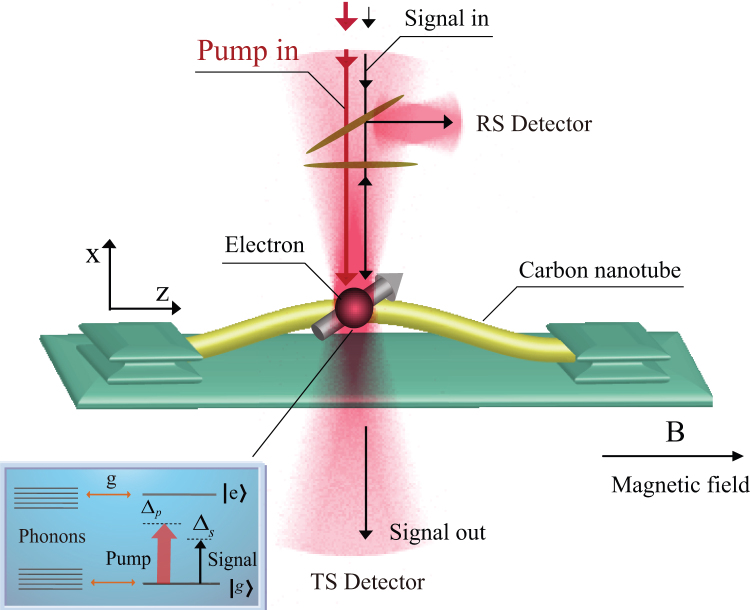
Schematic diagram of the detection of spin-based optomechanics with a carbon nanotube, where an electron spin is trapped in the center of nanotube in the presence of a strong pump field and a weak signal field. The continuous signal beam may be a single-photon source. The reflected signal (RS) and transmitted signal (TS) are detected by the specific apparatus. The left bottom window shows the energy levels of electron spin while dressing with the vibrational modes of carbon nanotube resonator.

The electron with spin trapped inside the center of CNT is shown in Fig. 1. For illustration of the numerical results, here we use the realistic experimental parameters^5^,^6^,^25^, Δ*_so_* = 0.37 *meV*, *L* = 500 *nm*, *l*_0_ = 2.5 *pm*, *κ* = 0.05 *MHz*, Γ_1_ = 0.1 *MHz* and *ω_p_*/2*π* = 500 *MHz*, and we find the coupling strength between the electron spin and the nanotube vibrational mode *g*/2*π* = 0.56 *MHz*. In the following, based on the coupled spin and nanotube system, we propose a method to determine the spin-orbit coupling strength, as well as two designs of a single photon router and a quantum microwave transistor.

### Determination for spin-orbit coupling strength

**Figure 2:**
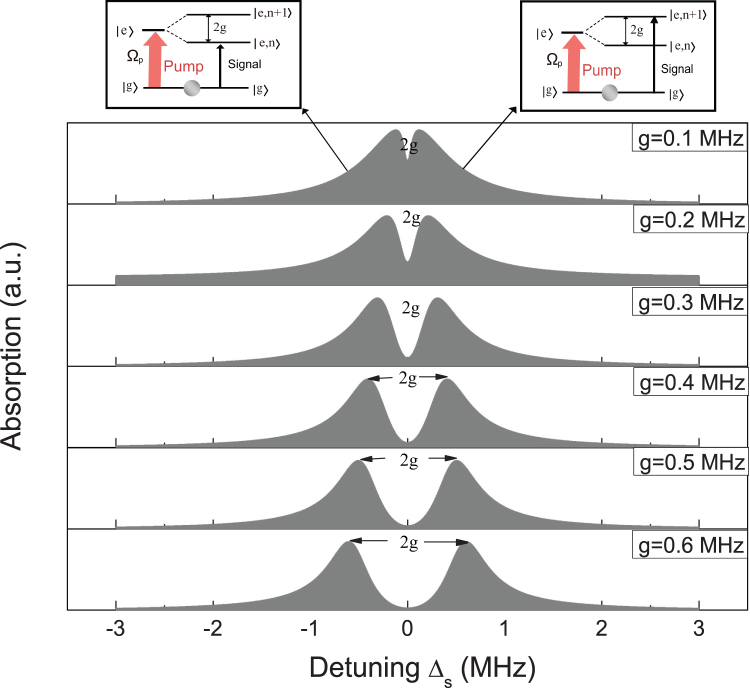
The plot of the signal absorption spectrum as a function of detuning Δ*_s_* with different coupling strength *g* = 0.1, 0.2, 0.3, 0.4, 0.5, 0.6 *MHz*. The left inset and right inset represent the energy level transitions of the left peak and right peak appeared in the spectrum, respectively. The parameters used are *κ* = 0.05 *MHz*, Γ_1_ = 0.1 *MHz* and *ω_p_*/2*π* = 500 *MHz*^5^,^6^,^25^, Δ*_p_* = Δ*_n_* = 0 and 
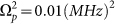
.

While radiating a strong pump field and a weak signal field on this coupled spin-CNT system, we plot the signal absorption spectrum. Fig. 2 shows the absorption spectrum of the signal field (the imaginary part of the dimensionless susceptibility *Im_χ_*^(1)^) as a function of the signal-spin qubit detuning Δ*_s_* (Δ*_s_* = *ω_s_* − *ω_q_*) with different coupling strength *g*. Since dressing with the vibrational modes of carbon nanotube, the upper level of electron spin |*e*〉 splits into |*e*, *n*〉 and |*e*, *n*+1〉. The two peaks displaying in Fig. 2 for a given coupling strength represent the spin-orbit interaction. As shown in the insets of Fig. 2, the left peak signifies the transition from |*g*〉 to |*e*, *n*〉, while the right peak is represented by the |*g*〉 → |*e*, *n* + 1〉 transition (|*n*〉 denotes the phonon number states of the nanotube resonator). The splitting distance becomes larger with increasing the coupling strength, which can strongly reveal the spin-orbit coupling. For the qubit-resonator coupling, the coupling strength *g* has a simple relationship with the spin-orbit coupling strength Δ*_so_* via *g* = Δ*_so_l*_0_/*L*. From Fig. 2 we find that the splitting distance is exact twice times larger than the spin-CNT coupling strength, which provides a straight way to measure the spin-orbit coupling in CNTs. For the bottom plot in Fig. 2, the splitting distance between two peaks is 1.2 *MHz*, which just corresponds to the coupling strength *g* = 0.6 *MHz*.

Furthermore, the signal absorption equals to zero at Δ*_s_* = 0 in Fig. 2, which means the input signal field is transmitted to the coupled system without absorption. Such a phenomenon is attributed to the quantum interference between the vibration modes (phonon modes) and the beat of the two optical fields via the spin. If the beat frequency of two lasers *δ* = *ω_s_* − *ω_p_* is close to the resonance frequency *ω_n_* of the CNT, the nanotube resonator starts to oscillate coherently, which results in Stokes (*ω_s_* = *ω_p_* − *ω_n_*) and anti-Stokes (*ω_as_* = *ω_p_*+*ω_n_*) scattering of light from the CNT via the electron spin. The Stokes scattering is strongly suppressed because it is highly off-resonant with the spin frequency. However, the anti-Stokes field can interfere with the near-resonant signal beam and thus modify the signal beam spectrum. Here the CNT resonator plays a vital role in this coupled system so that we can refer the above phenomenon as phonon induced transparency, which is analogous with electromagnetically induced transparency in atomic systems^13^.

### A single photon router

**Figure 3:**
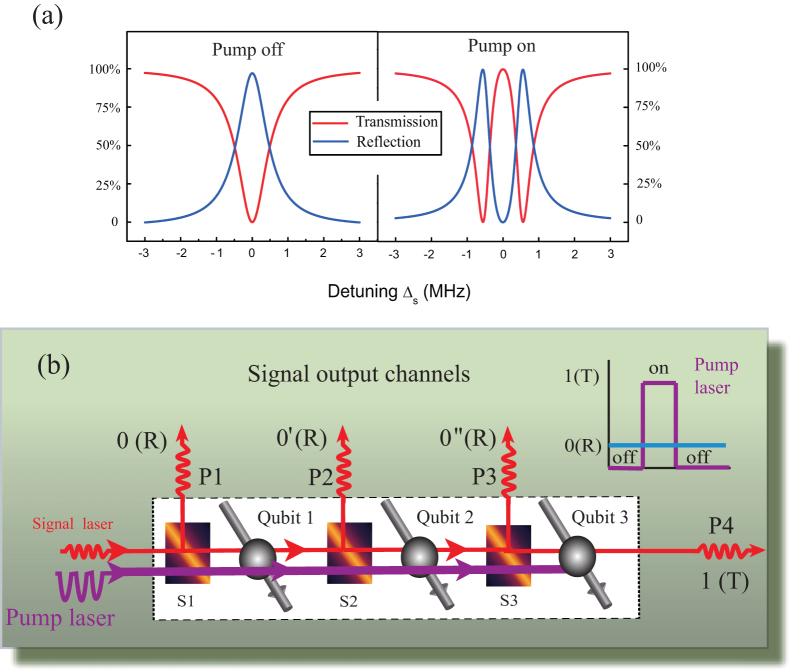
(a)The transmission and reflection spectrum of a signal beam with and without the strong pump field.The parameters used are (*κ*, Γ_1_, *ω_n_*, *g*, Δ*_p_*, Δ*_n_*) = (0.05, 0.1, 500, 0.56, 0, 0)*MHz*^5^,^6^,^25^, and 
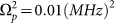
 (b) The operation scheme for multi-output single photon router channels, using three electron spin qubits, labeled as Qubit1, Qubit2 and Qubit3. When inputting a weak signal filed in the single photon regime at *ω_s_*, whether the output channels (P1, P2, P3, P4) can receive the signal is decided by the pump field on and off status. The path between the reflection output port and the transmission output port can be achieved by simply turning off and on the pump field, respectively. Inset: the pump pulse sequence.

According to the phonon induced transparency, we can design a single photon router by the spin-based optomechanical system with carbon nanotubes. Fig. 3(a) shows transmission spectra and reflection spectra of the signal field as a function of the detuning Δ*_s_*, respectively. In the absence of the pump field, the left plot in Fig. 3(a) exhibits a standard Lorentzian shape and an inverted Lorentzian shape in the reflection and transmission spectra of the signal field, which signifies the completely reflected signal beam through the coupled spin-CNT, where the mechanical oscillation of the CNT has no effect to the propagation of the signal beam. However, as the pump beam turns on and fixes the detuning Δ*_p_* = Δ*_n_* = 0, the reflection spectrum and the transmission spectrum present a completely different behavior, as shown the right plot in Fig. 3(a). From the figure, we can see that the transmission is 100% but the reflection is zero at the resonance. That is to say, when pumping the coupled system with a suitable pump laser power, the resonant signal beam will be transmitted completely while the reflection is totally suppressed, which is opposite to the case of pump beam absence.

Due to the fact that the reflection and the transmission of the signal beam can be controlled effectively by the pump beam, here we propose a protocol for implementing a single photon router based on this coupled spin-CNT system, as shown in Fig. 3(b). We input a weak resonant signal in the single photon regime to the electron spin system, and then fixing the pump field on resonance with spin qubit and CNT resonator (Δ*_p_* = Δ*_n_* = 0). When turning off the pump field, the signal photon completely reflect from the electron to the RS (reflected signal) detector as shown in Fig. 1. Otherwise, if turning on the pump field, the signal photon will directly transmit the electron to the TS (transmitted signal) detector. We should note that such a single photon router also works well in the microwave regime^26^ and at an ultralow power of the pump field, which is required for a practical spin-router used in quantum information networks. As shown in Fig. 3 (a), the Rabi frequency of the pump field in this single photon router is as low as 0.01(*MHz*)^2^, which corresponds to 0.2 *pW*. Furthermore, the switching time of this router is determined by the electron spin dephasing time, which can be controlled in actual experiments. Therefore, this spin-optomechanical router can also operate at high speed and efficiency. Fig. 3(b) shows the operation scheme of the router for multi-channels where we can embed three spin qubits in the same CNT resonator.

### A quantum microwave transistor

**Figure 4:**
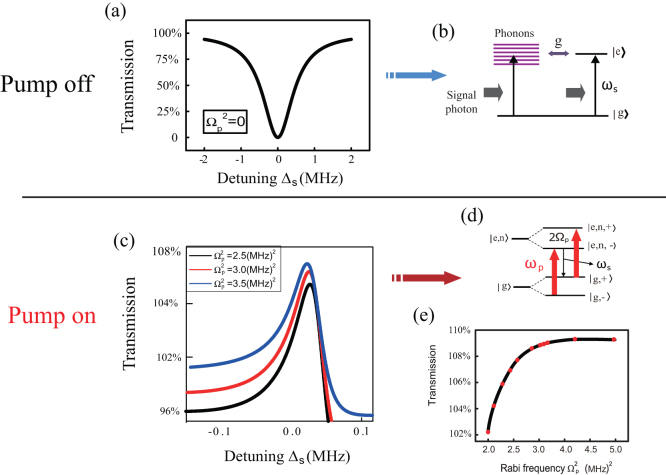
The transmission spectrum for the coupled spin-CNT, which can be used as a quantum microwave transistor. The parameters used here are (*κ*,Γ_1_,*ω_n_*,*g*, Δ*_p_*, Δ*_n_*) = (0.05,0.1,500,0.56,2,−2) MHz^5^,^6^,^25^. (a)The attenuation of the signal field as a function of its frequency detuning from the electron spin, in the absence of pump field; (b)The transition of the absorbed signal field shown in (a); (c)When turning on the pump beam, the amplification of the signal beam enlarges as the power of the pump beam increases; (d)The energy level transitions of three-photon process which amplifies the signal beam in (c); (e) The characteristic curve of the quantum microwave transistor by plotting the gain values for the transmission of the signal beam as a function of the Rabi frequencies of pump field.

Furthermore, because of the spin frequency *ω_p_* can be regulated by the external magnetic field, one can achieve a quantum mirowave transistor based on this coupled spin-CNT system by fixing the detuning Δ*_p_* = −Δ*_n_*. Fig. 4 displays a series of transmission spectra of the signal field as a function of detuning Δ*_s_* for various Rabi frequencies of pump field, where Δ*_p_* = −Δ*_n_* = 2 *MHz*.

Fig. 4(a) shows the transmission spectrum of the signal field in the absence of pump field, which is the usual Lorentzian line shape of the bare electron spin. This plot shows that in the absence of the pump beam, the system attenuates the weak signal beam totally, which arises from the usual absorption resonance, as shown in Fig. 4(b). However, as the pump beam turns on, the dip becomes a peak immediately (Fig. 4(c)). As the pump power increases even further, one can observe more amplification of the signal field, which is the result of the increasing feed of photons into the electron spin qubit. This pump beam, like a switch, dramatically controls the transmission of the signal beam. These curves in Fig. 4(c) demonstrate that the spin-optomechanical system can indeed act as a microwave transistor, where the output signal (‘source’) field can be regulated by the input pump (‘gate’) field. This amplification behavior is caused by quantum interference between the dressed states while applying two fields. Fig. 4(d) shows the origin of this three-photon resonance physical process. Here the electron makes a transition from the lowest energy level | *g*, –〉 to the dressed level | *e*, *n*, +〉 by the simultaneous absorption of two pump photons and emission of a photon at Δ*_s_* = 0, as indicated by the region of amplification of the signal beam in Fig. 4(c). It is clearly seen that transmission is greatly enhanced around the detuning Δ*_s_* = 0 at the higher pump power. For more specific description, we further investigate the transistor characteristic behavior of the coupled spin-CNT by plotting the amplification of signal beam as a function of the Rabi frequencies of pump field as shown in Fig. 4(e). The transmitted signal field can be amplified abruptly and greatly after the pump power reaches a critical value, which indicates an excellent transistor action. Therefore, the transmitted signal field can be attenuated or amplified in this coupled system under the control of the pump field by fixing the detuning Δ*_p_* = −Δ*_n_*. Besides, as discussed above, the switching time in quantum microwave transistor is obviously tunable by the dephasing time of electron spin.

## Discussion

In this paper, we theoretically investigate spin-based optomechanics with carbon nanotubes, using a pump field and a signal field. We show that the spin-orbit coupling strength can be determined by the peak splitting distance in signal absorption spectrum, while fixing the pump field on resonance with the frequencies of spin qubit and carbon nanotube. Due to the phonon induced transparency, we further propose two protocols of a single photon router and a quantum microwave transistor, which operate at ultralow pump power (~pW) and tunable short switching time. These nanoscale router and transistor presented here will offer potential applications in scalable spin-based quantum networks and quantum information processing.

## Methods

Our design is based on an electron trapped in a suspended carbon nanotube, under the radiation of a strong pump field (with frequency *ω_p_*) and a weak signal field (with frequency *ω_s_*), as shown in Fig. 1. The vibrational modes of nanotube resonator can be treated as phonon modes. In the presence of a high magnetic field **B**, it constitutes a well-defined twolevel spin system (TLS) with an effective spin-phonon coupling due to the interplay between SOI and flexural vibrations of the nanotube^15^,^16^,^17^. The energy splitting 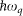 of the TLS is tunable with the magnetic field. The inset window of Fig. 1 shows the two-level spin state, while dressing with the flexural modes of CNT via the spin-phonon coupling. The coupling strength *g* depends on the vibrational amplitude as well as the spin-orbit coupling strength. Recently, Rudner and Rashba^18^ have studied the spin and valley dynamics on a nominally four-fold degenerate orbital level of a carbon nanotube, as well as the coupling of electron spin to the bending modes of CNT, where the spin-phonon coupling mechanism is presented. They indicated that at long phonon wavelengths the deflection coupling is dominated, while at short wavelengths the deformation potential coupling should be dominated.

For simplicity we consider only the deflection coupling mechanism, but note that the approach can readily be extended to include both effects. The Hamiltonian describing this system is^17^,^18^
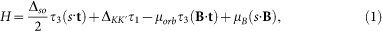
where Δ*_so_* represents the curvature enhanced spin-orbit coupling, which leads to a spin polarization along the nanotube axis. The inter-valley scattering induced by lattice impurities and characterized by Δ*_KK′_*, hybridizes the two valley (isospin) degrees of freedom^19^. *τ_i_* and *s_i_* are the Pauli matrices in valley and spin space, **t** is the tangent vector along the CNT axis. Additionally, the external magnetic field, **B** = *Be_z_*, applied along the axis of the unbent CNT, gives rise to an orbital and spin Zeeman effect through *µ_B_* and *µ_orb_*.

We next study how the spin qubit couples to the quantized mechanical motion of the CNT. In the presence of a strong confinement potential, the ground state multiplet of TLS is nearly fourfold degenerate, which can be described by the states |*sτ*〉 of spin (*s* ∈ {↑↓}) and isospin (*τ* ∈ {*K*,*K*′}). Then we consider only a single polarization of flexural motion (along the x-direction), assuming that the two-fold degeneracy is broken, e.g., driven by external electric fields^7^. A generic deformation of the CNT with deflection *u*(*z*) makes the tangent vector **t**(*z*) coordinate-dependent. Expanding **t**(*z*) with small deflections, the coupling terms *s* · **t** and **B** · *t* in Hamiltonian (1) can be expressed as 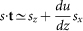, 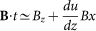. The deflection function *u*(*z*) can be described as 
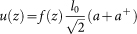
, where *a*^+^ and *a* are the creation and annihilation operators for a quantized flexural phonon mode, respectively. *f*(*z*) and *l*_0_ are the waveform and zero-point amplitude of the phonon modes, respectively. Under the radiation of a strong pump field and a weak signal field, the system Hamiltonian described the coupled electron spin and carbon nanotube can be written as *H* = *H*_0_ + *H_int_* + *H_drive_* with^7^

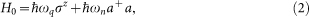

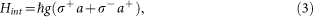

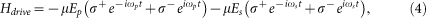
where *H*_0_ denotes the Hamiltonian of TLS and CNT respectively, where 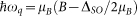. *ω_n_* is the vibrational frequency of the nanotube resonator. *H_int_* represents their coupling with the coupling constant *g* = Δ*_so_l*_0_/*L*, where *L* is the length of the CNT. When applying a strong pump field with amplitude *E_p_*, and a weak signal field with amplitude *E_s_*, the TLS coupled to the radiation fields can be described as Hamiltonian term *H_drive_*, where *µ* is the transition diploe moment of the electron. In the rotating frame at the pump field frequency *ω_p_*, the total Hamiltonian can be expressed by
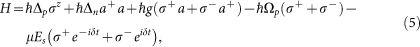
where Δ*_p_* = *ω_q_* − *ω_p_* and Δ*_n_* = *ω_n_* − *ω_p_* are the frequency detunings between spin qubit-pump field and CNT-pump field, respectively, *δ* = *ω_s_* − *ω_p_* is the difference between signal field and pump field, Ω*_p_* is the Rabi frequency of the pump field and is given by 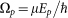.

Applying the Heisenberg equations of motion for operators *σ^z^*, *σ*^−^ and *a* and introducing the corresponding damping and noise terms, we derive the quantum Langevin equations as follows^20^,^21^:
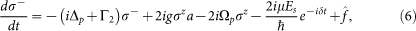

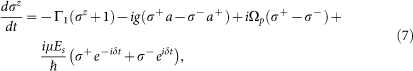

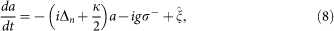
where Γ_1_ and Γ_2_ are the TLS spontaneous emission rate and dephasing rate, respectively, *κ* is the decay rate of CNT^22^, 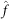 is the *δ*-correlated Langevin noise operator, which has zero mean 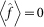 and obeys the correlation function 
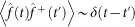
.

The motion of carbon nanotube resonator is affected by thermal bath of Brownian and non-Morkovian process^20^,^23^. The quantum effects on the resonator are only observed in the limit of high quality factor, that obeys 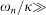. The Brownian noise operator can be modeled as Markovian with the decay rate *κ* of the resonator mode. Therefore, the Brownian stochastic force has zero mean value 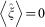 that can be characterized as^23^ 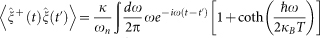. Following standard methods from quantum optics, we derive the steady-state solution to Eqs. (6)–(8) by setting all the time derivatives to zero. They are given by 
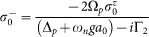
, 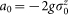, where 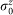 is determined below using equation (14). To go beyond weak coupling, we can always rewrite each Heisenberg operator as the sum of its steady-state mean value and a small fluctuation with zero mean value as follows: 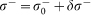, 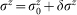, *a* = *a*_0_ + *δa*. Inserting these equations into the Langevin equations Eqs. (6)–(8), one can safely neglect the nonlinear term *δaδσ*^−^. Since the driving fields are weak, but classical coherent fields, we will identify all operators with their expectation values, and drop the quantum and thermal noise terms^8^. Then the linearized Langevin equations can be written as:
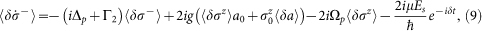

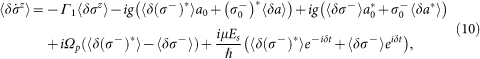

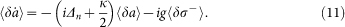
In order to solve Eqs. (9)–(11), we make the ansatz^24^ 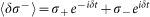, 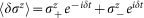 and 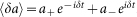. Upon substituting these equations to Equations (9) – (11), and working to the lowest order in *E_s_* but to all orders in *E_p_*, we can obtain *σ*_+_, which corresponds to the linear susceptibility as follows: 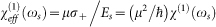, where *χ*^(1)^(*ω_s_*) is given by
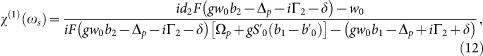
where 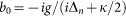, 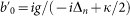, 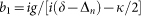, 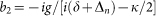, 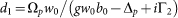, and 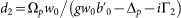,
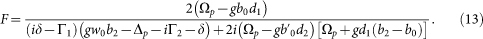
The population inversion (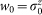) of the electron is determined by the following equation:
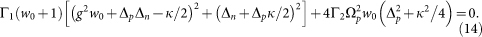


## Author Contributions

J.J. finished the main work of this article, including deducing the formulas, plotting the figures, and drafting the manuscript. K.D. conceived of the idea, participated in its writing and provided some useful suggestions. All authors read and approved the final manuscript.
